# Looking beyond efficacy: The implementation science exploration of the Australian PITCH study, a national stepped‐wedge cluster RCT evaluating a dementia training program for home care workers

**DOI:** 10.1002/alz.085017

**Published:** 2025-01-09

**Authors:** Anita M Y Goh, Ellen Gaffy, Lidia Engel, Yared Belay, Sanne Peters, Christa Dang, Colleen Doyle, Steven Savvas, Frances Batchelor, Lee‐Fay Low, David Ames, Susan Malta, Claudia Cooper, Gill Livingston, Anita Panayiotou, Samantha M Loi, Anne Fairhall, Cathy Roth, Briony Dow

**Affiliations:** ^1^ The University of Melbourne, Melbourne, VIC Australia; ^2^ National Ageing Research Institute, Melbourne, VIC Australia; ^3^ Edith Cowan University, Perth, Western Australia Australia; ^4^ National Ageing Research Institute, Parkville, VIC Australia; ^5^ La Trobe University, Melbourne Australia; ^6^ Monash University, Melbourne, VIC Australia; ^7^ The University of Melbourne, Parkville, VIC Australia; ^8^ Swinburne University, Melbourne, VIC Australia; ^9^ The University of Sydney, Sydney, NSW Australia; ^10^ University of Melbourne, Melbourne, VIC Australia; ^11^ Queen Mary University of London, London United Kingdom; ^12^ University College London, London United Kingdom; ^13^ Victorian Department of Health, Melbourne, VIC Australia; ^14^ University of Melbourne, Parkville, VIC Australia; ^15^ Neuropsychiatry, The Royal Melbourne Hospital, Parkville, VIC Australia; ^16^ Family carer, Melbourne, VIC Australia; ^17^ PALZ ‐ Professionals with Alzheimer’s and related diseases, Geelong, VIC Australia

## Abstract

**Background:**

The Promoting Independence Through quality Care at Home (PITCH) project aimed to improve outcomes for people with dementia and their carers via a co‐designed training intervention for home care workers (HCWs). The results of the primary efficacy analysis of the successful stepped‐wedge cluster RCT (n = 172 HCWs in 18 clusters in 7 Australian service providers) were presented at AAIC 2023.

**Method:**

This presentation goes beyond efficacy and discusses the implementation science (process evaluation and behavioural change) and health economic analysis of the intervention.

**Result:**

Process Evaluation: Many intervention trials only focus on specific outcomes, with little consideration of the processes behind intervention effectiveness. However, solely focusing on outcomes in intervention trials without exploring the underlying processes limits understanding of what works, and hinders knowledge translation. Process evaluation allows the consideration of the mechanisms of change or lack of change. We present the process evaluation of the PITCH intervention, barriers and facilitators to implementation, and factors that related to unexpected problems and variation in intervention delivery, using the Theoretical Domains Framework of behaviour change.

Behaviour Change Techniques (BCTs): Linking BCTs with theories of behaviour change allows investigation of mechanisms underlying intervention effects, and extends process evaluations. To understand how interventions may result in behaviour change, this presentation reports our findings of BCTs used in all published RCTs in HCWs (including PITCH), using the Behaviour Change Technique Taxonomy (v1). Results presented include robust evidence synthesis about which BCTs are effective (and ineffective) within HCW interventions and how they vary as a function of contextual variables (e.g. setting, population).

Health economics: This presentation reports the economic evaluation of the PITCH intervention, adopting a cost‐consequences analysis discussing whether PITCH provides good value for money. We found that integrating health economics provided additional key information, ensuring efficient use of resources, benefiting care‐recipients and healthcare systems.

**Conclusion:**

Using newly‐analyzed results from a large RCT in HCWs, this presentation goes beyond the traditional efficacy results of RCTs and reports on the importance, methods, and results of analyzing other key intervention characteristics: 1) process of intervention delivery, 2) effective (and ineffective) behavioural change techniques, and 3) health economic analysis.

